# Data on the uptake and metabolism of the vertebrate steroid estradiol-17β from water by the common mussel, *Mytilus* spp.

**DOI:** 10.1016/j.dib.2016.10.030

**Published:** 2016-11-05

**Authors:** Tamar I. Schwarz, Ioanna Katsiadaki, Benjamin H. Maskrey, Alexander P. Scott

**Affiliations:** Centre for Environment, Fisheries and Aquaculture Science, Barrack Road, Weymouth, Dorset DT4 8UB, UK

**Keywords:** Mollusc, Oestradiol, Sulphate, Steroid metabolism

## Abstract

The data presented in this article primarily provide support for the research article entitled “Mussels (*Mytilus* spp.) display an ability for rapid and high capacity uptake of the vertebrate steroid, estradiol-17β from water” (T.I. Schwarz, I. Katsiadaki, B.H. Maskrey, A.P. Scott, 2016) [1]. Data are presented on the ability of mussels to absorb tritiated estradiol (E_2_) from water. The data indicate that most of the radioactivity remaining in the water is 1,3,5(10)-estratriene-3,17β-diol 3-sulfate (E_2_ 3-S) and the radioactivity in the mussel tissue is mainly in the form of fatty acid esters. The latter, following saponification, were identified by ultra-high performance liquid chromatography in conjunction with tandem mass spectrometry (UHPLC-MS/MS) as intact E_2._ Data are included that indicate that the remaining radioactivity in the tissue is composed of E_2_ 3-S and unidentified free metabolites. Experimental data included also relate to a) the efficiency of extraction of radioactivity from tissue, b) the efficiency of separation of free and esterified E_2_ using solvents and c) possible factors affecting the recovery of radioactivity. Finally, preliminary data are provided on concentrations of immunoreactive E_2_ in the free and ester fractions of tissue extracts from mussels caged in the field.

**Specifications Table**

**Table:** 

Subject area	*Biology*
More specific subject area	*Endocrinology*
Type of data	*Figures and Tables*
How data was acquired	*Scintillation counting, HPLC, thin layer chromatography (TLC), Radioimmunoassay*
Data format	*Raw and analyzed*
Experimental factors	*Studying the rate of uptake of tritiated E*_*2*_*by live mussels, developing a new method for separating free and esterified steroids in tissue extracts, identifying the metabolites*
Experimental features	*Measuring the rate of disappearance of tritiated E*_*2*_*from water in vessels containing mussels, extracting and then separating the metabolites in both water and tissue by liquid/liquid partition and by chromatography, definitively identifying metabolites by tandem mass spectrometry using a Waters Xevo TQ mass spectrometer*
Data source location	*The Retreat, Brancaster Staithe, Norfolk; Menai Strait, Wales; Portland Harbour, Dorset*
Data accessibility	*Data presented in this article*

Value of the data

• The data provide supporting evidence to challenge the assumption that E_2_ found in the flesh of mollusks is of endogenous origin.
• Identification of E_2_ 3-S in water and tissue is essential for understanding E_2_ uptake and metabolism in *Mytilus* spp.
• The development of a new and simple way to separate free and esterified E_2_ from tissue extracts will help those working on the origin and role (if any) of vertebrate steroids in mollusks.

## 1 Data

**Fig. 1:**
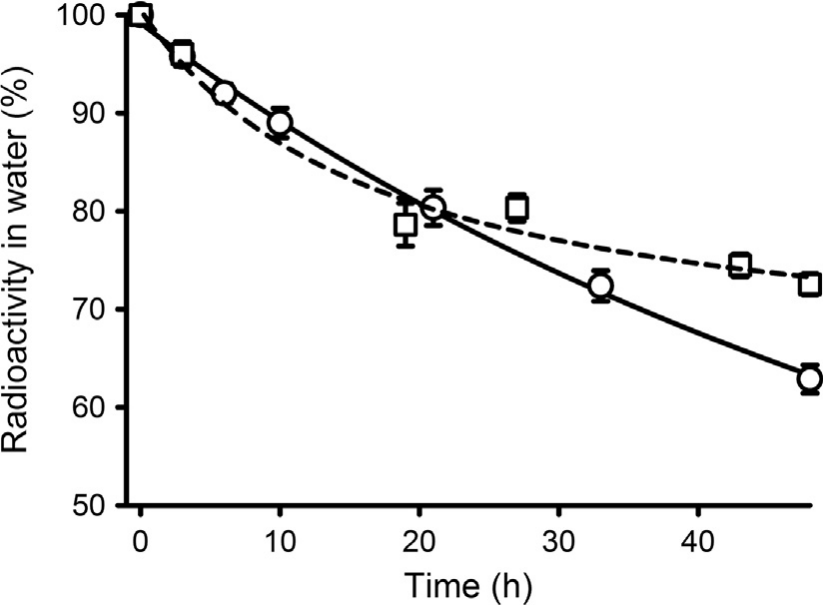
Removal of radiolabel from water by *Mytilus* spp. (Experiment 1) exposed for two consecutive 48 h periods (1st=○; 2nd=□) under the following conditions – glass tanks, 13 L seawater tank^−1^, 5 animals tank^−^^1^, 0.7 µCi L^−1^ (1.36 ng L^−1^) [^3^H]-E_2_, water change. Data are presented as mean percentage±S.E.M. of radiolabel remaining in the water (*n*=10 tanks per time point). The lines show the best fit of the data for each period (1st, solid; 2nd, dash) to a three parameter hyperbolic decay equation. A single sorption control with radiolabel but no animals was included for each exposure period, but as there was no evidence for any losses in the 1st period, no adjustments were made to the data.

**Fig. 2:**
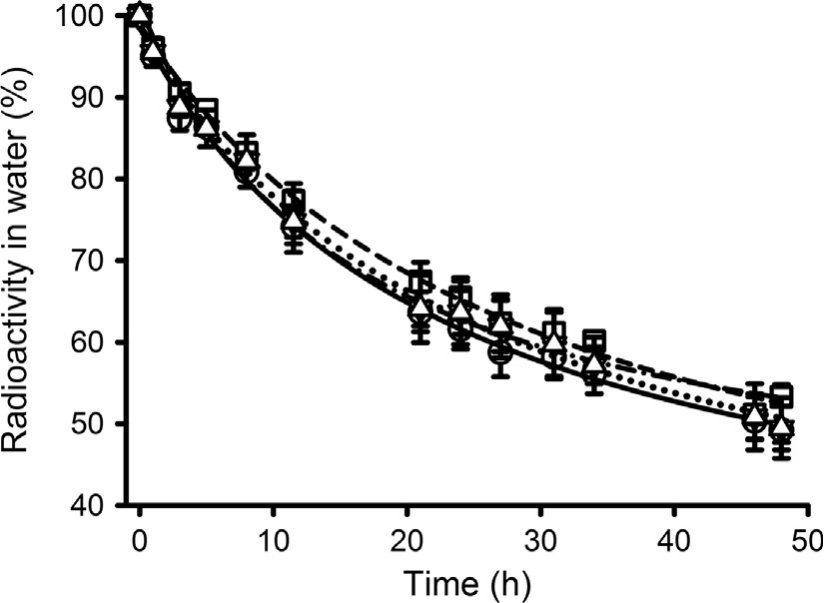
Removal of radiolabel from water by *Mytilus* spp. (Experiment 2) in the presence of food (▼) and low and high concentration of cold E_2_ (7.1 ng L^−1^ E_2_, ☐ and 35.7 ng L^−1^ E_2_: △) compared with a [^3^H]-E_2_-only treatment (O) during a 48 h exposure under the following conditions – glass tanks, 7 L seawater tank^−1^, 5 animals tank^−1^, 0.7 µCi L^−1^ (1.36 ng L^−1^) [^3^H]-E_2_. Data are presented as mean percentage±S.E.M. of radioactivity remaining in the water (*n*=3 tanks). The lines represent the same data. ([^3^H]-E_2_-only, solid; feed, dot-dash; low cold, dotted; high cold, dash) fitted to a three parameter hyperbolic decay equation. A single sorption control with radiolabel but no animals and two feed sorption controls with radiolabel and algae but no animals were included, but as there was no evidence for any losses, no adjustments were made to the data.

**Fig. 3:**
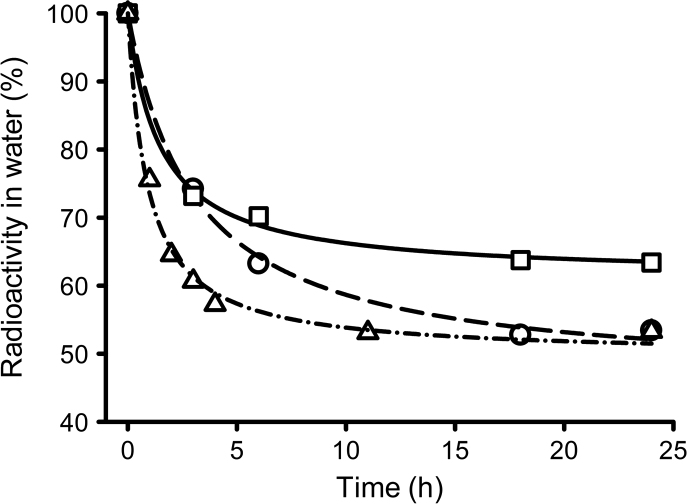
Removal of radiolabel by *Mytilus* spp. during three separate 24 h exposures to [^3^H]-E_2_. Two of the exposures (Experiments 4 & 5; ○, □) were carried out in duplicate with aeration and 5 animals in 2 L water (they were set up as positive controls to examine the uptake of other steroids). The final exposure (Experiment 6; △) was carried out in a single vessel with 18 animals in 3.6 L water (and was set up for a subsequent depuration experiment). The lines represent the same data fitted to a three parameter hyperbolic decay equation.

**Fig. 4:**
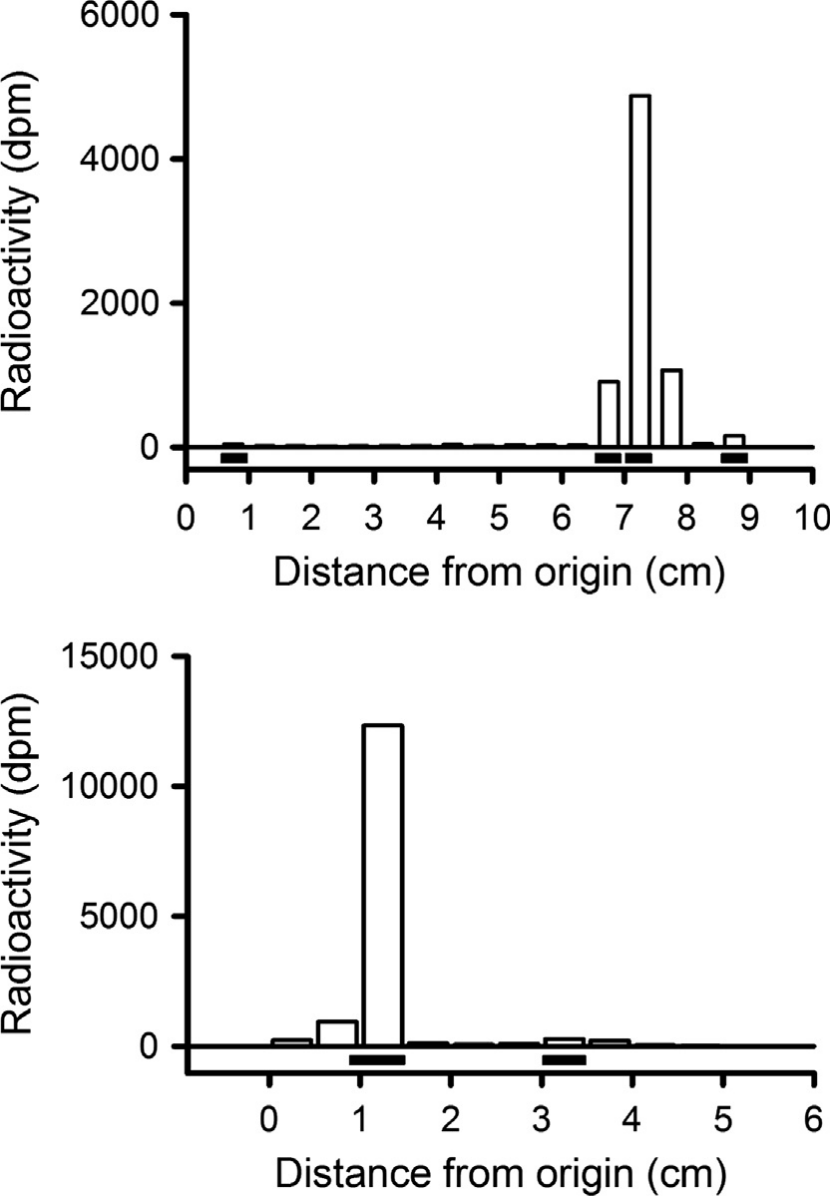
Thin layer chromatographic separation of a putative sulfate peak (fraction 38) obtained via reverse phase HPLC separation of water that had been collected from mussels exposed to [^3^H]-E_2_ for 24 h (Fig. 2 in [1], original water sample from Experiment 4 and 5). Top graph: pattern of separation of radioactivity without any treatment; standards (horizontal black bars under the x axis) were run concurrently, from left to right: cortisol glucuronide, E_2_ 17β-S, E_2_ 3-S and E_2_. Bottom graph: pattern of separation of radioactivity after removal of the sulfate group with sulfatase; E_2_ (black bar on the left) and estrone (black bar on the right) standards were run concurrently. NB. The two TLC separations were run at separate times with different mobile phases.

**Fig. 5:**
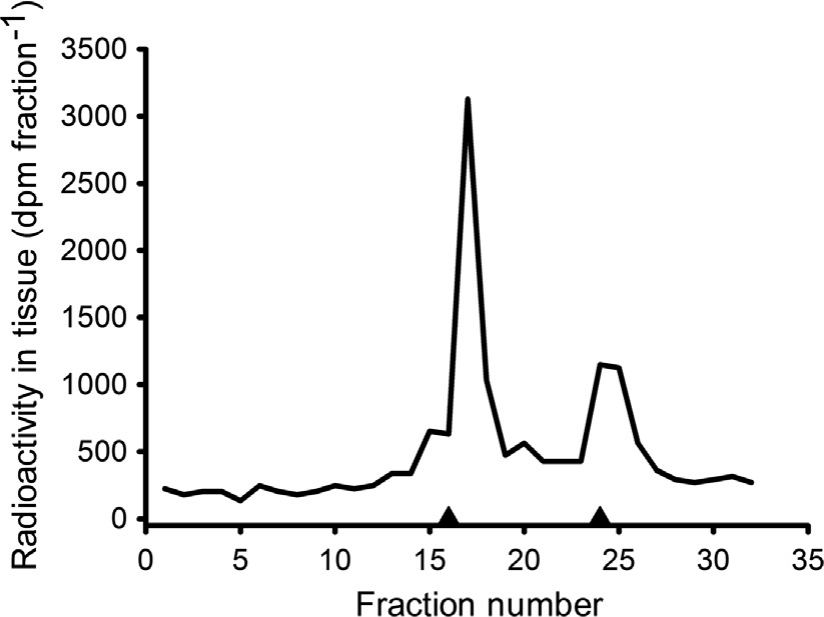
Chromatography on a reverse phase HPLC preparative column of [^3^H]-E_2_ radioactivity derived from the 80% ethanol (free and sulfate) fraction of a pooled mussel extract (from Experiment 6). Data are presented as radioactivity (solid line) and UV absorption at 280 nm of E_2_17β-S and E_2_ standards that were run concurrently (▲; from left to right).

**Fig. 6:**
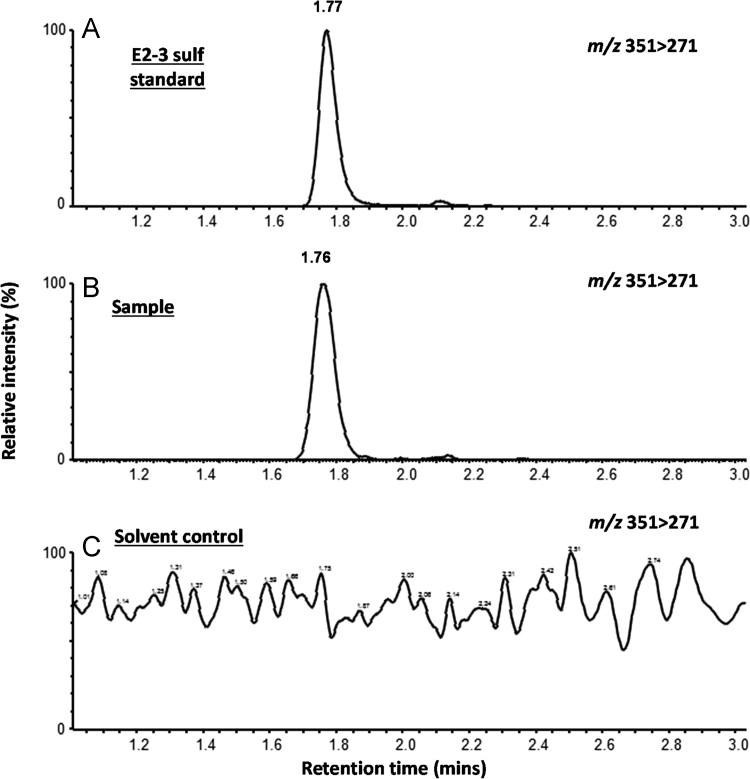
Identification of E_2_ 3-S in 80% ethanol fraction of tissue extract from five mussels exposed to cold E_2_ for 24 h (Experiment 7). Representative UHPLC-MS/MS chromatograms of a negative ion MRM transition of 351>271 of authentic E_2_ 3-S standard (panel A), 80% ethanol fraction from E_2_-treated mussel extract (panel B) and 80% ethanol fraction from solvent control treated mussel extract (panel C).

**Fig. 7:**
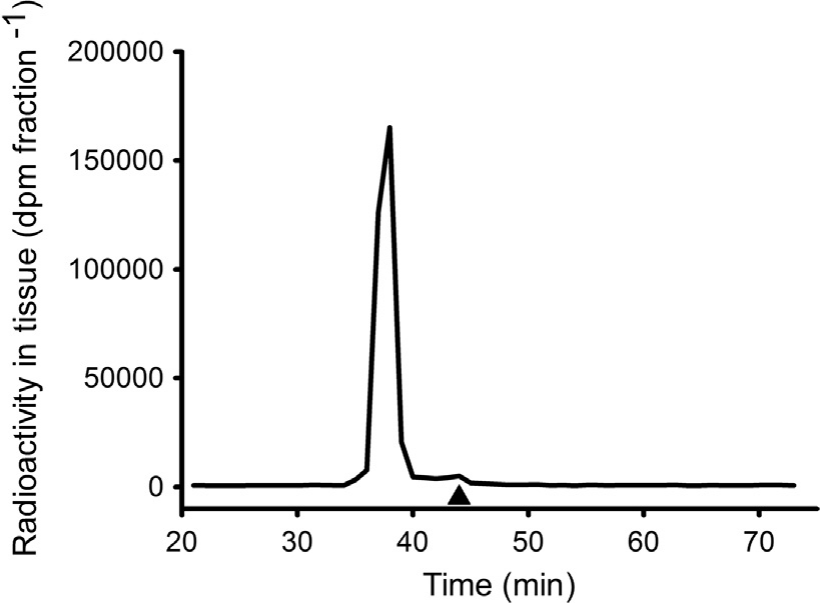
Normal phase HPLC chromatogram of tissue extract from mussels that had been exposed to [^3^H]-E_2_ for two consecutive 48 h periods (animals from Experiment 1). Data are presented as radioactivity (solid line) and UV absorption at 280 nm of E_2_ standard that was run concurrently (▲).

**Fig. 8:**
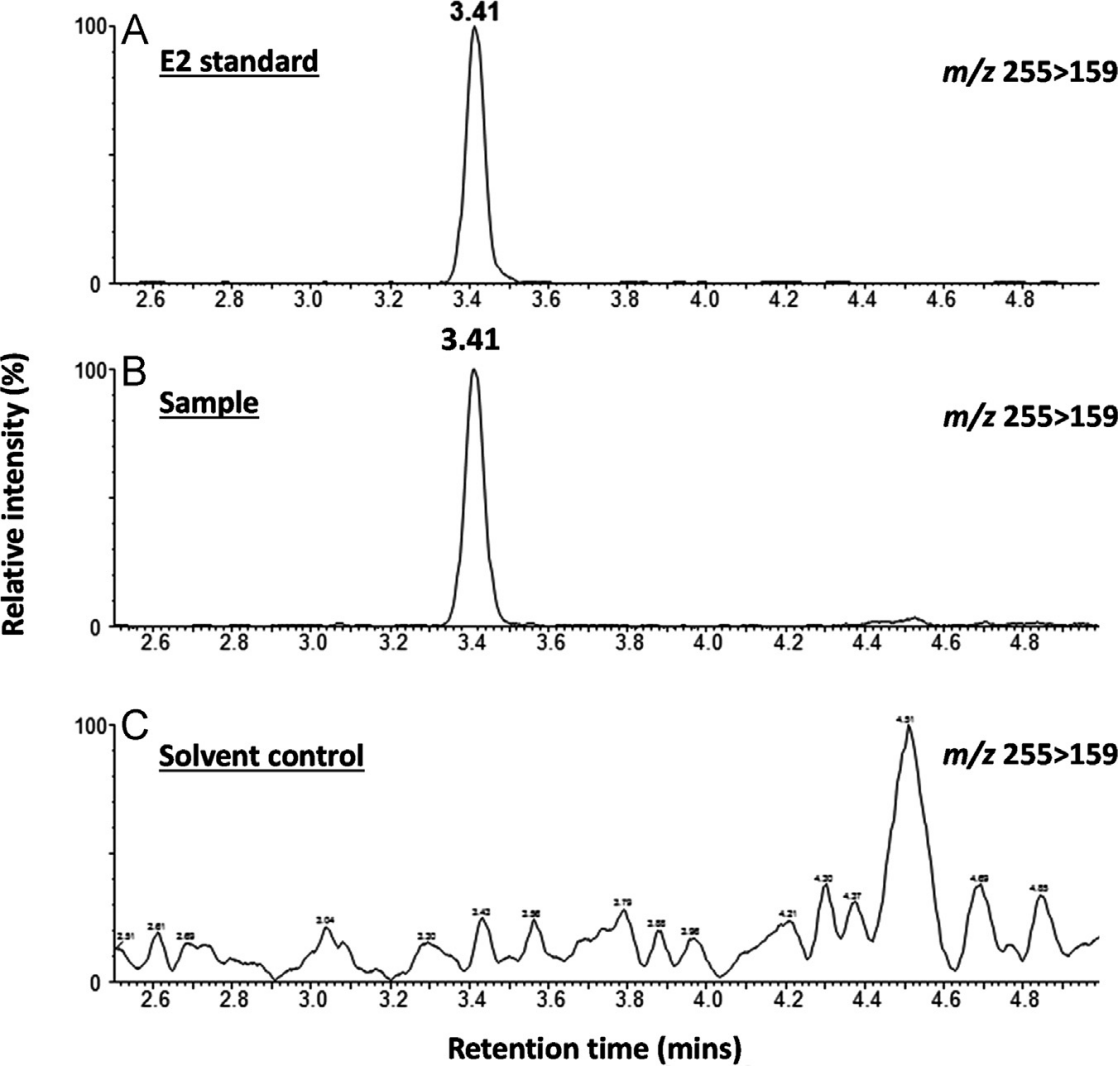
Identification of E_2_ in the saponified heptane fraction (hydrolysate) of tissue extract from five mussels exposed to cold E_2_ for 24 h (Experiment 7). Representative UHPLC-MS/MS chromatograms of a positive ion MRM transition of 255>159 of authentic E_2_ standard (panel A), hydrolysate from E_2_-treated mussel extract (panel B) and hydrolysate from solvent control treated mussel extract (panel C).

**Fig. 9:**
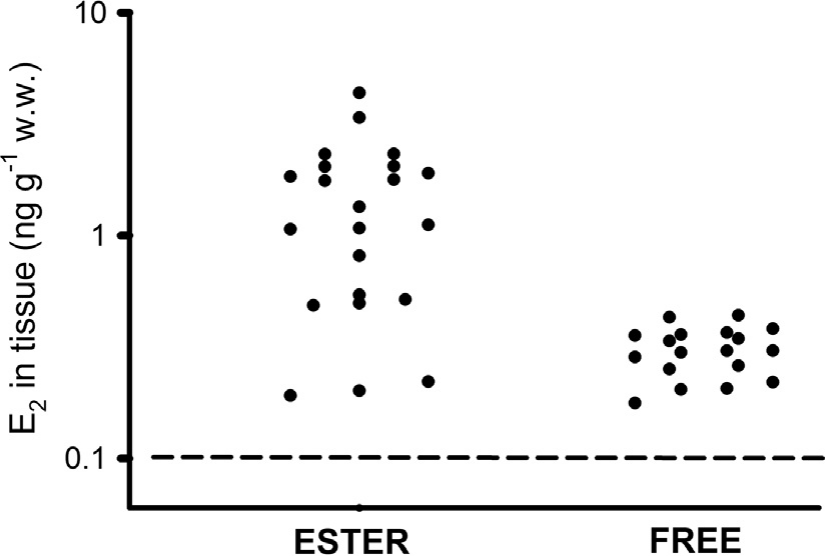
Immunoactive E_2_ concentrations (ng g^−1^ wet weight) in the ester and free fractions of twenty mussels caged at three sites in the Thames estuary, an offshore site and a reference site. The horizontal dotted line indicates the detection limit of the radioimmunoassay.

The data presented in this article show the uptake of radiolabeled E_2_ ([^3^H]-E_2_) from water by mussels under different conditions (Fig. 1, Fig. 2, Fig. 3); the production of E_2_ metabolites in water (E_2_ 3-S; Fig. 4) and tissue extracts (E_2_ 3-S, Fig. 5, Fig. 6; E_2_ esters, Fig. 7), the identification of intact E_2_ in the ester fraction of saponified tissue extracts (Fig. 8) and the concentrations of free and esterified E_2_ in tissue extracts of mussels caged in the field (Fig. 9). Original data from experiments that were carried out to determine the best methods for extracting (Table 1) and then separating free and esterified E_2_ (Table 2) are also presented.

## 2 Experimental design, materials and methods

### 2.1 Laboratory exposures of mussels to [^3^H]-E_2_

A summary of the exposure conditions (including controls) for the experiments described below are in Table 1 of the research article [1]. All mussels (*Mytilus* spp.) were acclimatized prior to exposure for at least 1 day, where they were fed Shellfish Diet^®^ 1800 (unless stated otherwise) following manufacturer׳s instructions and the water was changed daily. The time at which water samples (1 mL) were taken during exposure for scintillation counting are indicated in the relevant Figures.

#### 2.1.1 Experiment 1

Mussels were obtained from The Retreat, Brancaster Staithe, Norfolk - a catchment which holds a class B shellfish harvesting classification – in March 2014. Animals were glued to glass rods (later considered to be unnecessary) and placed vertically in aerated cylindrical glass tanks at 16±1 °C with a 16:8 h light:dark photoperiod for exposure to [^3^H]-E_2_ (Fig. 1). The water was changed after 48 h and the radioactivity refreshed. After exposure, mussel soft tissue was extracted with Method 1 (Table 1) for putative identification of [^3^H]-E_2_ lipid soluble metabolites using normal phase HPLC (Fig. 7).

#### 2.1.2 Experiment 2

Mussels were obtained from ‘Deepdock Mussels’ in the Menai Strait, Wales – a catchment which also holds a long term class B shellfish harvesting classification. The animals were harvested in April 2014, transported in a cool-box overnight and immediately placed in a flow-through system of seawater. Animals were suspended (in nets) in aerated glass tanks at 16±1 °C with a 16:8 h light:dark photoperiod for exposure to [^3^H]-E_2_ in the presence of food or cold (unlabeled) E_2_ (Fig. 2). The water was changed daily and the animals in the feeding treatment were fed a combination of live algae (*Isochrysis* spp. and *Tetraselmis* spp.) three times a day at a concentration of 95 cells µL^−1^. The amount of feed required was calculated as the equivalent to 2.5% of mean expected mussel dry weight and the concentration was based on the range of 50–100 cells µL^−1^. The ratio *Isochrysis*:*Tetraselmis* was 1:3 in order to achieve the appropriate mass and concentration.

#### 2.1.3 Experiment 3

Mussels were collected from Portland Harbour, Dorset in May 2014. The nearby northeast Portland Harbour breakwater is a catchment holding a long term class B shellfish harvesting classification. The data for this exposure (for which temperature was not controlled and ranged between 17.7–21.2 °C) are presented in the research article [1].

#### 2.1.4 Experiments 4 and 5

Mussels were collected from Portland Harbour in October 2014. Animals were placed in a bucket lined with a polyethylene bag with aerated seawater at 16±1 °C with a 16:8 h light:dark photoperiod for exposure to [^3^H]-E_2_ (Fig. 3). Water (50 mL) samples were taken at 24 h and extracted [1] for examination of [^3^H]-E_2_ metabolites (see Section 2.3 and Fig. 4).

#### 2.1.5 Experiment 6

Mussels were collected from Portland Harbour in November 2015 and placed in a bucket lined with a polythene bag with aerated seawater for exposure to [^3^H]-E_2_ (Fig. 3). After exposure, mussel soft tissue was extracted using Method 2 (Table 1) and separated (see research article [1] and Table 2 for method optimization) for identification of putative water soluble [^3^H]-E_2_ metabolites by HPLC (Fig. 5).

#### 2.1.6 Experiment 7

Mussels were collected from Portland Harbour in May 2015 and placed in beakers lined with polythene bags with aerated seawater for exposure to cold E_2_. Animals were then extracted (Method 1) and separated for identification of both lipid soluble metabolites in the saponified ester fraction (heptane phase) and water soluble metabolites (in the 80% ethanol phase) by UHPLC-MS/MS (Fig. 8 and Fig. 6 respectively).

### 2.2 Mussels caged in the field

Mussels (*Mytilus* spp.) were kindly provided by Dr Tim Bean of the Cefas Laboratory (with thanks to the Port London Authority, The Historic Dockyard and Chatham and Southend Council for allowing the placement of cages). The mussels were originally collected from Morston shellfishery in Norfolk (reference site) and depurated for two weeks before being deployed for eight weeks in cages in three locations in the Thames (Gravesend, Southend-on-Sea and Chatham) and one at an offshore site (Wharp). The animals were delivered to the laboratory in a cool-box and stored at −20 °C; four animals from each site (20 in total) were processed. Mussel soft tissue was extracted (Method 1) and separated into free, ester and sulfate fractions with the same method employed for radioactive residues [1]. The ester fraction was then subjected to saponification and the sulfate fraction to acid solvolysis as described in [1]. All three fractions were then reconstituted in radioimmunoassay (RIA) buffer for subsequent quantification of immunoactive E_2_ concentrations using RIA [2] (Fig. 9).

### 2.3 Thin layer chromatography

A portion of the major HPLC peak found in water at 24 h (presumptive sulfated [^3^H]-E_2_; see Fig. 2 in [1]) was mixed with 10 µg each of E_2_ 3-S, estradiol 17β-sulfate (E_2_ 17β-S), T glucuronide and E_2_ and loaded as described previously [3] onto one lane of a TLC plate (catalog no. LK6DF; Whatman Labsales; www.whatman.com; but no longer manufactured). Standards were also separately loaded onto adjacent lanes. The plate was developed for 45 min with a mixture of ethyl acetate:ethanol:ammonia solution (45:45:15, v-v:v), which enables not just free, but also sulfated and glucuronidated steroids to migrate on the silica gel [3] (Fig. 4, top graph). The plate was sprayed with 10% phosphomolybdic acid in ethanol and heated at 150 °C for 5 min to display the positions of the standards. Lanes were then divided into 5 mm bands (ensuring that the two sulfates, that ran close together, were in different bands) and the silica gel from each band was scraped off the plate. The scraped gel bands were mixed with 500 μL ethanol, 500 μL water and 7 mL scintillation fluid and placed in the scintillation counter. Free [^3^H]-E_2_ metabolites were also separated on a TLC plate but using chloroform:ethanol (98:2, v-v) as a mobile phase (Fig. 4, bottom graph).

### 2.4 Experiments to investigate factors affecting recovery of radioactivity from tissue extracts

a) Color quenching? All tissue extracts had an orange color (of varying intensity). The efficiency of color-quench correction (an option provided by the manufacturers of the scintillation counter) was tested by selecting a heavily colored extract from a mussel that had not been exposed to radiolabel. A range of volumes of extract (0, 0.1, 0.25, 0.5, 1, 2 and 3 mL) were placed in duplicate into scintillation vials and left to evaporate overnight. The dry extracts were then mixed with an identical amount of radioactivity (*c*. 50,000 dpm), 0.5 mL of water and 7 mL scintillation fluid and counted using color quench correction. The extracts had a mean+S.E.M. of 52,769+2208 dpm (coefficient of variation=11%; *n*=7 in duplicate).
b) Adsorption to mussel shells? Empty shells from Experiment 6 were washed twice in diethyl ether. The solvent was decanted directly into a scintillation vial, dried, reconstituted in 1 mL of 80% ethanol and 7 mL scintillation fluid and counted. The amounts of radioactivity adsorbed to shells were insignificant (mean±S.E.M. 1±0.15% of the total radioactivity in the soft tissue).
c) Tritiated water in the extracts? To test this, nine tissue extracts were counted with and without drying them before adding scintillation fluid. No differences were found between extracts (mean 10,596 vs 10,133 dpm; *n*=9) that had been counted with and withoutdrying (i.e. there was no evidence for any tritiated water in tissue extracts).

## Figures and Tables

**Table 1:** Steroid extraction (from tissue) procedure development.

Step		Action	Radioactivity recovery (mean %±SD)
**Method 1 (*n*=5)**			
1	10 mL methanol	Grind/shake	47.1±30.1
2	5 mL methanol	Shake	22.9±7.9
3	3 mL methanol:chloroform (1:2, v-v)	Shake	16.1±11.2
	After 3 steps		**86.1**±15.4

**Method 2 (*n*=8)**			
1	8 mL methanol:ethyl acetate (3:5, v-v)	Grind/shake	87.0±6.7
2	5 mL ethyl acetate	Shake	10.6±5.6
	After 2 steps		**97.6**±1.3

NB. The volumes are per animal (not per gram of wet tissue)

**Table 2:** Steroid separation procedure development.

Optimization steps	Sample	Mixing time (min)	Mussel extract vol. (µl)	Water vol. (ml)	Ethanol vol. (ml)	Heptane vol. (ml)	Ethanol activity (%)	Heptane activity (%)
Initial separation: free v. ester	Free	5	400	0.3	1.2	1.5	98.6	1.4
	Ester	5	400	0.3	1.2	1.5	21.8	78.2
Does extract amount and heptane volume have an effect? Amount: No Volume: Yes (up to 3 mL)	Ester	5	100	0.3	1.2	1.5	28.9	71.1
	Ester	5	100	0.3	1.2	3	22.0	78.0
	Ester	5	100	0.3	1.2	4.5	21.0	79.0
	Ester	5	200	0.3	1.2	1.5	28.9	71.1
	Ester	5	200	0.3	1.2	3	21.1	78.9
	Ester	5	200	0.3	1.2	4.5	20.8	79.2
	Ester	5	400	0.3	1.2	1.5	27.7	72.3
	Ester	5	400	0.3	1.2	3	21.7	78.3
	Ester	5	400	0.3	1.2	4.5	19.9	80.1
Is more than one heptane extraction beneficial? Yes	Ester	5	200	0.3	1.2	1.5	13.8	63.4
						1.5		15.9
						1.5		4.6
						1.5		1.5
						1.5		0.7
Does increasing the proportion of ethanol improve separation? No	Ester	5	200	0.3	1.2	1.5	14.8	72.8
						1.5		8.8
						1.5		3.6
	Ester	5	200	0.15	1.35	1.5	19.3	57.8
						1.5		14.6
						1.5		8.3
Does increase in mixing time improve extraction? No	Purified ester	5	200	0.3	1.2	3	4.2	82.7
						3		13.1
	Purified ester	10	200	0.3	1.2	3	4.3	81.2
						3		14.4
	Purified ester	15	200	0.3	1.2	3	4.0	87.7
						3		8.3
Does extract amount have an effect? No (up to 800 µl).	Purified ester	5	400	0.3	1.2	3	2.6	84.9
						3		12.5
	Purified ester	5	600	0.3	1.2	3	2.2	85.5
						3		12.3
	Purified ester	5	800	0.3	1.2	3	2.2	86.2
						3		11.6
						4.5		8.7
